# Robotic-Assisted Epicardial Hybrid Ablation and Left Appendage Closure in Persistent Atrial Fibrillation: First European Experience

**DOI:** 10.3390/jcm13061563

**Published:** 2024-03-08

**Authors:** Alfonso Agnino, Laura Giroletti, Ascanio Graniero, Piersilvio Gerometta, Matteo Parrinello, Giovanni Albano, Eduardo Celentano, Ernesto Cristiano, Giuseppe Nasso, Natasja M. S. de Groot

**Affiliations:** 1 Department of Cardiovascula Surgery, Division of Robotic and Mini-Invasive Cardiac Surgery, Humanitas Gavazzeni-Castelli, 24125 Bergamo, Italy; alfonso.agnino@gavazzeni.it (A.A.); laura.giroletti@gavazzeni.it (L.G.); ascanio.graniero@gavazzeni.it (A.G.); piersilvio.gerometta@gavazzeni.it (P.G.); 2 Department of Cardiac Anesthesia, Humanitas Gavazzeni-Castelli, 24125 Bergamo, Italy; matteo.parrinello@gavazzeni.it (M.P.); giovanni.albano@gavazzeni.it (G.A.); 3 Cardiac Electrophysiology Complex Operational Unit, Humanitas Gavazzeni-Castelli, 24125 Bergamo, Italy; eduardo.celentano@gavazzeni.it (E.C.); ernesto.cristiano@gavazzeni.it (E.C.); 4 Cardiology Department, Erasmus University Medical Center, 3015 GD Rotterdam, The Netherlands; n.m.s.degroot@erasmusmc.nl; 5 Faculty of Health, Medicine and Life Sciences, Maastricht University, 6211 LK Maastricht, The Netherlands

**Keywords:** atrial fibrillation, robotic surgery, left appendage closure

## Abstract

**Background:** Pulmonary vein isolation is currently considered to be the gold standard for ablating paroxysmal atrial fibrillation. However, its efficacy is limited in patients with persistent atrial fibrillation. The convergent procedure has emerged as a hybrid ablation. This study aims, for the first time in the literature, to introduce a hybrid approach that includes epicardial ablation with cutting-edge robotic technology and subsequent electrophysiological study to verify and an endocardial ablation to complete the ablation lines. **Methods:** We present 18 cases of robotic-assisted epicardial hybrid ablation performed between April and December 2023 on patients with long-standing persistent atrial fibrillation (mean age: 64 ± 5 years; mean duration: 4 ± 2 years). All of the procedures were performed at “Humanitas Gavazzeni Hospital”, Bergamo, Italy. Robot-assisted epicardial ablation performed using the “Epi-Sense AtriCure” device was guided by monitoring electrogram morphology and point-by-point impedance drop. This approach also included left atrial appendage occlusion and the disconnection of the ligament of Marshall. An electrophysiological study and endocardial ablation were planned three months after the procedure. **Results:** The procedure was successfully executed in all patients with no major complications and a mean operative time of 142 ± 22 min. None of the cases required conversion to full sternotomy or minithoracotomy. The procedure was performed in all cases without extracorporeal circulation and on a beating heart. Fifteen patients (83%) were extubated in the operating room. The length of stay in the intensive care unit was less than 24 h. Acute restoration of sinus rhythm was achieved in 12 out of the 18 patients (67%); the median duration of their hospital stay was two days. In the electrophysiological study, seven pts had sinus rhythm, two had atrial fibrillation, and one patient developed atrial flutter at 3-month follow-up. Patients underwent transcatheter ablation to complete the lesion set and, at the time of discharge, were all in sinus rhythm. **Conclusions:** In our initial experience, surgical atrial fibrillation ablation consisting of a unilateral thoracoscopic technique facilitated by a robotic platform and continuous EGM monitoring has proven to be safe and feasible. For the electrophysiological study at 3 months, completing the gaps in the surgical ablation lines could improve the clinical results of the technique in terms of sinus rhythm stability. However, mid- and long-term follow-up is required to demonstrate this.

## 1. Introduction

Atrial fibrillation is the most common adult arrhythmia, with a prevalence of 2–4%. The delineation between paroxysmal and persistent atrial fibrillation remains the most common classification. However, it must be noted that these forms are not always independent of each other. This classification is used in terms of making therapeutic decisions, which implies an attempt to restore sinus rhythm and not just rate control of the heart [1,2,3,4]. 

Currently, a patient’s symptoms and quality of life are placed at the center of interest when choosing a treatment. Atrial fibrillation, in fact, is a pathology that has very high variability in terms of symptomatology: cases can range from totally asymptomatic to patients with highly disabling symptoms. Electrical isolation of the pulmonary veins (PVIs) has emerged as the primary therapy for atrial fibrillation (AF) [5], which established the role of pulmonary vein (PV) ectopy triggering AF. However, in the case of long-standing persistent AF, the post-PVI AF recurrence rate remains high and increases with longer intervals between the initial AF episode and the ablation procedure [6,7]. Furthermore, over the years, the left atrial appendage (LAA) and the ligament of Marshall (LOM) have also been identified as contributing to the arrhythmogenesis of atrial fibrillation and, therefore, potential targets for ablative therapy.

Suboptimal outcomes of catheter ablation (CA), particularly in the subset of patients with more persistent types of AF, have driven the pursuit of alternative methodologies. 

Since its inception in 1987, the Cox maze surgical procedure for the treatment of persistent atrial fibrillation has proven effective, maintaining sinus rhythm in 80–90% of patients [6].

The surgical treatment of AF can be divided into three procedures: concomitant to other cardiac surgical procedures, stand-alone (specifically for the treatment of only AF), or hybrid (followed by catheter-based ablation or vice versa).

However, despite the success rate, the invasiveness of the technique and higher morbidity compared with a catheter-based approach has limited its use, especially in cases of isolated disease.

Advancements in energy sources and devices have facilitated the development of minimally invasive closed-chest thoracoscopic epicardial pulmonary vein isolation [8,9,10,11], as described by several authors in recent years, in an attempt to replicate the efficacy of the Cox maze procedure, further reducing morbidity. To further evolve the technique, we have performed a mono-lateral thoracoscopic AF surgical ablation with robotic assistance. 

The main goal of the procedure is the possibility of creating a set of lesions that includes pulmonary vein and posterior atrial wall isolation plus LAA exclusion and LOM disconnection by exploiting the potential of the robotic platform.

In this scenario, the concept is that of a hybrid AF ablation approach that has evolved to amalgamate the benefits of thoracoscopic surgical ablation (TSA), providing direct visualization of the epicardial target sites, enabling the preservation of the anatomical structures and endocardial CA, enabling the verification of the conduction block across lesions, and leading to the creation of lesions in areas that are inaccessible to TSA. 

Recent reports underscore favorable mid-term AF freedom after a hybrid ablation procedure [12]. 

In addition, there are promising data that support the thoracoscopic or subxiphoid/transdiaphragmatic hybrid ablation approach [13]. 

In this study, we evaluate the safety, feasibility and preliminary results of robotic-assisted epicardial hybrid ablation and LAA closure in cases of persistent AF. 

## 2. Material and Methods

### 2.1. Study Population 

This study is a single-center retrospective registry. Eighteen patients affected by persistent AF underwent robotic-assisted epicardial surgical ablation and LAA closure through left thoracoscopy from April to December 2023 at Humanitas Gavazzeni Hospital in Bergamo, Italy, using the Da Vinci^®^ X Surgical System (software version n° P10- ntuitive Surgical Inc., Sunnyvale, CA, USA) and Episense device (Atricure, Mason, OH, USA). 

Three months after robotic-assisted epicardial hybrid ablation and LAA closure, all patients were evaluated with an electrophysiologic study (EPS) and treated with very high-power short-duration (vHPSD) ablation (Biosense Webster, Irvine, CA, USA). 

All of the clinical data were retrospectively extrapolated by our general and cumulative registry database (containing clinical information of all patients admitted to the hospitals) and then retrospectively analyzed. This study conforms to the ethical principles of the Good Clinical Practice and the Helsinki Declaration and is in compliance with the current regulations. All of the patients provided written informed consent for inclusion, the collection/use of data or samples, and/or publication according to the actual guidelines.

This retrospective observational paper was sent for notification to the ethics committee of IRCCS Humanitas Clinical Institute Rozzano, Italy (Prot. 32/23 GAV 19 September 2023).

### 2.2. Preoperative Diagnostic Workup

Prior to the procedure, computed tomography, CT, (third-generation 192-slice dual-source SOMATOM Force, Siemens Healthineers, Erlangen, Germany) and magnetic resonance imaging, MRI, (1.5 T MR-scanner Acheva, Philips Healthcare, Eindhoven, The Netherlands or Aera, Wan Chai, Hong Kong, Siemens Healthineers, Erlangen, Germany) are commonly performed to delineate the complex relationships between the left atrium, pulmonary veins, and surrounding structures. 

Radiological cardiac imaging has been described as an important tool in terms of guiding surgical and percutaneous procedures. It also provides information about chest wall anatomy to facilitate port placement. 

MRI was used to avoid multiple contrast injections, providing 3D anatomical imaging of LA and evaluating left atrial fibrosis with late enhancement assessment. Cardiac CT scan was adopted for patients where MRI was contraindicated.

Preoperative management included transthoracic or transesophageal echocardiography, electrocardiography, and routine biochemical procedures. 

### 2.3. Surgical Technique

A double-lumen endotracheal tube was placed for selective lung ventilation. Transesophageal echocardiography (TEE) was performed routinely to rule out left atrial appendage thrombosis. An esophageal probe (S-Cath Esophageal Temperature Probe, Circa Scientific, Englewood, CO, USA) utilizing seven temperature recording points continuously monitored endoluminal temperature during ablation to avoid thermal damage, ensuring that the temperature remained below a maximum threshold of 37.2 °C.

Unipolar, suction-assisted radiofrequency ablation was executed using a 3 cm self-irrigated Episense probe (Atricure Inc., Mason, OH, USA). The device integrates radiofrequency (RF) energy, direct visualization, and impedance monitoring to realize continuous lesions. RF energy was delivered at a pre-set level of 30 W for 90 s to create epicardial transmural lesions.

To maintain consistent contact between the Episense device and the wall of the left atrium, a vacuum seal was established. To prevent overheating of the device during the ablation process, saline solution was perfused continuously for cooling. 

The Episense probe was equipped with sensing electrodes that facilitate the direct transmission of electrograms to a commercially available recording system. This setup allowed for pacing, sensing, and recording directly from the device, enhancing the accuracy of lesion assessment. To facilitate this functionality, the Episense device was connected to the CardioTek recording system (software version number n° EPTRACER V1.0.5.12) using a CSK-2030 cable (Atricure Inc., Mason, OH, USA) and a 50 Hz pass filter. Atrial electrograms were continuously monitored by a physician electrophysiologist. The primary desired outcome was a steady and continuous drop in impedance. If there was persistence of the atrial electrograms or if the impedance drop fell short of the desired level (<10 Ohm), a second application of RF energy was administered.

To start the procedure, the patient was positioned in a supine position, with a square pad strategically located behind the left scapula to moderately raise the left hemithorax. 

The surgeon marked the third, fifth, sixth, seventh, and eighth intercostal space for guidance during port positioning. 

After left lung deflation, the first 8 mm port was placed in the third intercostal space, under the inferior border of the pectoralis, a major muscle. A second 8 mm port was positioned in the fifth intercostal space at the level of the mid-axillary line, while the third 12 mm port was inserted in the sixth or seventh intercostal space at the level of the posterior-axillary line. The last 8 mm port was placed in the seventh/eighth intercostal space at the anterior–mid axillary line level based on the patient’s anatomical conformation.

Carbon dioxide (CO_2_) insufflation was started through the third port with a target intrathoracic pressure of 8–10 mmHg. A robotic platform was placed on the right side of the patient, and the robotic arms were anchored to the designated working ports. A 30°endoscopic camera (Intuitive) was inserted into the second port in order to navigate the thorax, pericardium, and heart structures with enhanced views (10× magnification).

The surgeon identified and carefully opened the pericardium under the phrenic nerve; stitches were placed on the superior and inferior edge of the pericardium to suspend it and expose the cardiac structure. 

The left atrial appendage (LAA) was identified and gently moved to visualize and subsequently dissect the Marshall ligament.

The Epi-Sense device, inserted through the 12 mm port, was introduced into the pericardial space, and the first ablation line was performed on the left atrial roof, between the left and right superior pulmonary veins.

In the next step, the surgeon realized an ablation line connecting the superior and inferior left pulmonary veins: then he lifted the left atrium and, after identifying the superior vena cava, positioned the probe to create a third line on the left atrial floor.

A small hole was opened under the inferior edge of the pericardium incision. Through this, the probe was inserted to complete the ablation on the medial side of the confluence of the right pulmonary veins and the entire atrial posterior wall.

Finally, the surgeon completed the ablation procedure with the last two ablation lines, with the first located at the base of the left atrial appendage (LAA) and the second one located between the base and apex of the LAA. 

The surgical setup is visible in Figure 1. The complete set of ablation lines is visible in Figure 2, Figure 3, Figure 4, Figure 5, Figure 6 and Figure 7 and further depicted in Figure 8. In Figure 9, LAA closure is shown. 

During all of the robotic epicardial ablations, an electrophysiologist monitored atrial signals and tissue impedance drop. After the application, EGM voltage persisted, and additional applications were delivered. 

The robotic procedure ended with the sizing and placement of an epicardial clip (Atriclip Device AtriCure Inc., Mason, OH, USA) to occlude the left atrial appendage and prevent clot formation and subsequent cerebral vascular incidents. The cardiac anesthetist guided the clip’s positioning through TEE and controlled the complete closure of the LLA.

### 2.4. Statistical Analysis

Data are expressed as mean + standard deviation for the continuous variables and as a percentage for the categorical variables. 

## 3. Results

### 3.1. Study Population

Our initial experience involved 18 patients diagnosed with long-standing persistent AF, with an average duration of 4 ± 2 years. The mean age was 64 ± 5 years. None of the patients had previous cardiac or thoracic surgery. Table 1 summarizes the preoperative characteristics. Notably, 33% of the patients had previously undergone one or more electrical cardioversions, while all patients had made one or more attempts at pharmacological cardioversions and were receiving antiarrhythmic drugs, albeit without achieving effective rhythm control. Among these patients, seven (39%) had undergone prior transcatheter PVI, while the remaining eleven (61%) were de novo cases without a history of prior ablation procedures.

The anatomical findings are also listed in Table 1. Of note, in the MRI study, the left atrial volume was increased (156 ± 37.7 mL). All of the patients had normal left ventricular function on transthoracic echocardiographic evaluation. 

### 3.2. Intra- and Postoperative Results

The procedure was successfully performed in all patients without major procedural complications. The average number of energy applications per patient was 21 ± 6.

None of the patients required conversion to full sternotomy/minithoracotomy or cardiopulmonary bypass (CPB) implant. No major bleeding occurred that would have required surgical revision or blood transfusion. The intraoperative data are outlined in Table 2. After surgical ablation, electrical cardioversion was performed in patients with AF directly in the operating room with the restoration of sinus rhythm in all patients. The average duration of the procedure was 142 ± 22 min. In this study, 83% of patients were extubated in the operating room. The length of stay in the intensive care unit (ICU) was less than 24 h. A single drainage tube was positioned in the left pleural space and removed four to five hours after extubation. The mobilization of patients commenced following chest tube removal.

The median duration of hospitalization was 3 days [interquartile range: 2–3.5]. During this period, antiarrhythmic and anticoagulant medications were reintroduced. Continuous monitoring of heart rhythm was conducted, with only one case of new-onset atrial flutter being revealed for a patient. At discharge, sinus rhythm was achieved in 12 out of the 18 patients (67%). These six patients were previously subjected to electrical cardioversion before being discharged but without re-establishing sinus rhythm, so they went to the 3-month check-up. 

One patient experienced transient and asymptomatic diaphragmatic paresis, which fully resolved within the first week after the procedure, as documented after undergoing a chest X-ray.

**Figure 9:**
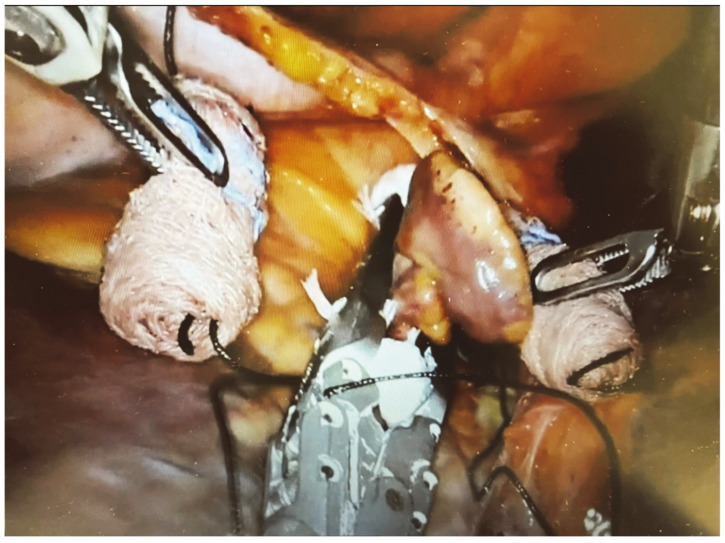
Robotic closure of the left atrial appendage.

Our protocol entailed a second stage at three months, during which patients underwent EPS and endocardial ablation. Of all patients, only 10 underwent the EPS study due to the short follow-up time. EPS demonstrated, through stimulation by CS, the transmission of the impulse to the right pulmonary veins that were reconnected, as illustrated in Figure 9. After completing right pulmonary vein isolation with vHPSD ablation (Biosense Webster, Irvine, CA, USA), patients maintained stable sinus rhythm until hospital discharge. During the EPS, only one patient presented with typical atrial flutter and underwent endocardial ablation of the mitral isthmus ablation through their coronary sinus. 

The esophageal probe (S-Cath Esophageal Temperature Probe, Circa Scientific) continuously monitored endoluminal temperature during ablation to avoid thermal damage, ensuring that the temperature remained below a maximum threshold of 37.2 °C, and this value was never exceeded during the procedures. 

## 4. Discussion

Atrial fibrillation is an important global public health problem due to the fact that it affects about 33 million people worldwide [14,15]. Despite surgical ablation (COX maze IV procedure) having demonstrated its efficacy, especially in persistent and long-standing persistent disease, invasiveness and operative risk reduce its use concomitantly with other cardiac surgical procedures [16]. 

Therefore, several minimally invasive techniques have been developed to replicate the results of the Cox maze procedure, avoiding cardiopulmonary bypass and reducing morbidity and complexity [17,18,19].

Possible approaches are minimally invasive surgery through the use of direct vision or in video thoracoscopy. 

In this heterogeneous scenario, we describe an alternative and even less invasive approach using a robotic platform to perform epicardial ablation, exploiting the important advantages of robotic instruments described in several papers [20,21] and minimizing surgical trauma on the chest wall.

Robotic tele-manipulators provide three-dimensional (3D) and magnified visualization. They are also equipped with articulating instruments that have seven degrees of freedom of motion, significantly optimizing dexterity. These features allow for the precise positioning and control of the ablation probe, especially at the level of the posterior left atrial wall, and better control of the surrounding cardiac structures, reducing the risk of damage. 

In our experience, despite the small number of patients treated with this technique in less than a year, we have exploited the surgical advantage of a robotic platform in terms of the visualization of ablation points, avoiding epicardial fat tissue, and ensuring the best conduction of RF. Ablation probe management has been improved through the use of robotic arms, with greater accuracy in terms of positioning and greater stability during energy delivery to ensure the complete transmural of the lesion. The procedure proved to be safe, without intraoperative complications requiring conversion at sternotomy, like cardiac structure damage or major bleeding (0%).

Most patients were extubated in the operating room (83%), and all patients were mobilized within 24 h of the procedure, leading to reduced hospitalization times (3 ± 1 days). 

All of the surgical ablation procedures were performed by a single surgeon skilled in cardiac robotic surgery, implying the importance and influence of the learning curve and experience on clinical outcomes.

Although it is still considered a cornerstone of persistent AF ablation, pulmonary vein isolation alone is not sufficient to maintain normal sinus rhythm in persistent forms of the disease. Therefore, several additional lesions have found clinical application [22,23]. Among these, growing interest has been focused on the ablation of the posterior atrial wall. Cardiac MRI has demonstrated a high prevalence of interstitial fibrosis in this area [24], and EP studies have shown the high prevalence of autonomic ganglionic plexi and macro-reentrant circuits that may also contribute to AF substrates [25,26]. Although the current results relating to DECAAF II do not provide evidence that cMR-guided fibrosis ablation improves AF recurrence in the setting of persistent AF, knowing the distribution and extension of fibrosis can help in terms of guiding decisions on the extension and orientation of the ablative lines to follow, improving mid- and long-term results [27].

The CONVERGE trial [28] describes, in the context of hybrid ablation (surgery plus EP), the creation of posterior wall epicardial lesions, in addition to other ablation lines, using the Epi-Sense device through subxiphoid access under thoracoscopic view.

In our technique, the posterior wall is isolated using the same probe but with access via a 12 mm port placed in the sixth or seventh intercostal space at the posterior–axillary line level, with only minor trauma to the chest. Potential thermal damage to the esophagus is avoided with constant temperature control through an esophageal tube, and we managed it with no related problems. 

The role of the LAA in increasing the risk of thromboembolism in these patients is known, but more recently, many studies have focused their attention on non-PV triggers as the source of AF, and within these, LAA represents one of the most common sites.

In the LAALA-AF (Left Atrial Appendage Ligation and Ablation for Persistent Atrial Fibrillation) registry [29], the collected data reinforce the relevance of the LAA in AF persistence and showed better results in terms of freedom from AF in the group that received both mechanical and electrical LAA isolation. Similar results are reported in the BELIEF (Effect of Empirical Left Atrial Appendage Isolation on Long-term Procedure Outcome in Patients With Persistent or Longstanding Persistent Atrial Fibrillation Undergoing Catheter Ablation) randomized trial [30]: LAA trans-catheter isolation improves long-term freedom from arrhythmias without increasing the number of complications.

Based on this evidence, we added the epicardial ablation of LAA and mechanical exclusion using the AtriClip device to our procedure. Proper clip placement is directly visualized, while TEE echocardiography confirms complete LAA closure. Several studies have demonstrated that the AtriClip achieves acute electrical isolation [31], in addition to thrombosis prevention, by reinforcing RF ablation lines. 

Finally, in our technique, the collaboration of different professional figures (surgeons, anesthetists, electrophysiologists, and cardiologist specialists in imaging) is fundamental. Teamwork allows for a more precise assessment of the patient in the preoperative period, better monitoring of the success of the surgical procedure intraoperatively, and the possibility of an endocardial ablation as a second step in the context of a hybrid strategy.

Several studies have proven the success of hybrid ablation. For example, in the CONVERGE Trial [28] at 12 months, freedom from AF was achieved in 67.7% of pts through the use of the hybrid convergent procedure and 50% via endocardial ablation. Van der Heijden and colleagues demonstrated in a randomized controlled trial that after 12 months, the freedom of AF off antiarrhythmic drugs was higher in the hybrid group compared with the catheter ablation group (89% vs. 41%) [32]. Nasso et al. demonstrated that the hybrid approach for the treatment of AF was safe and effective in immediately restoring sinus rhythm and in its maintenance at follow-up. The results showed that both immediate and staged procedures show similar efficacy, but this result is strongly influenced by the concomitant ablation of Bachmann’s bundle, which appears to be the most important component of the treatment strategy, in order to reduce the risk of recurrent AF [33].

In our cases, the single surgical procedure had encouraging results at the first step of follow-up in terms of the restoration of sinus rhythm. However, we performed the endocardial transcatheter study in just a few patients (10/18) due to the short follow-up time, and only one needed hybrid completion (10%) due to atrial flutter. These early pieces of evidence lead to a possible increase in the restoration of sinus rhythm in almost all patients with AF through the use of a hybrid approach. The high percentage of recurrence during the first days after the procedure (33%) can be accepted when considering the aim of resolving the arrhythmic situation at the end of all the therapeutic stages. 

The number of patients is obviously too small, and the follow-up is currently too short to provide statistically significant conclusions on clinical advantages over time. Our results are clearly speculative but also very promising and will be explored in further studies to confirm the evidence obtained so far. 

## 5. Limitations of the Study

There are limitations to the current study. Firstly, using a retrospective design introduces potential bias and limitations in data collection. Moreover, the absence of a control group hampers our ability to draw definitive conclusions regarding the effectiveness of the intervention.

Additionally, the small and very selective nature of the population cohort are the main limitations, even if nothing is known and published to the best of our knowledge in that kind of surgical population. 

Furthermore, the postoperative study is incomplete because not every patient underwent the second EP and ablation procedure. 

A prospective randomized trial is mandatory to confirm our results. 

Moreover, although the first effects are already visible after a few months in terms of sinus rhythm restoration, we could not capture the long-term effects in our population. We will continue to follow up on this population in the future to answer that limitation. However, the importance of our preliminary findings is maintained, notwithstanding the short follow-up time.

## 6. Conclusions

The initial experiences described in this study show that this novel unilateral thoracoscopic robotic-assisted AF ablation technique is safe and feasible. Although it may be very speculative, considering the limitations of the study, the hybrid strategy, including EP study and endocardial ablation at 3 months, could improve the clinical results of the technique in terms of RS stability and could be an important advancement in the treatment of persistent/long-standing persistent atrial fibrillation.

However, a larger sample of patients and long-term clinical follow-up are needed.

## Figures and Tables

**Figure 1:**
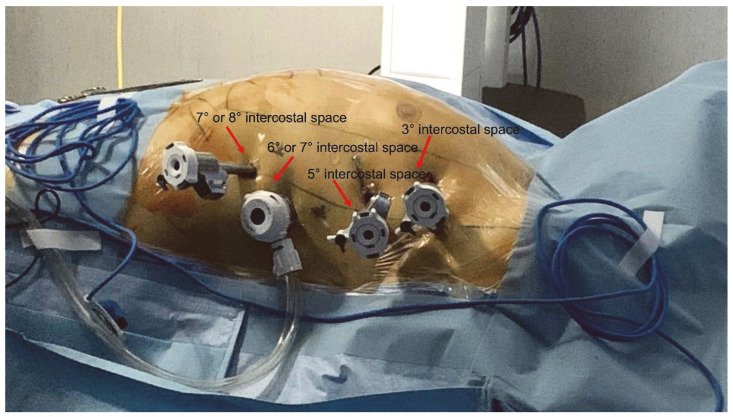
Working port assessment in robotic AF ablation.

**Figure 2:**
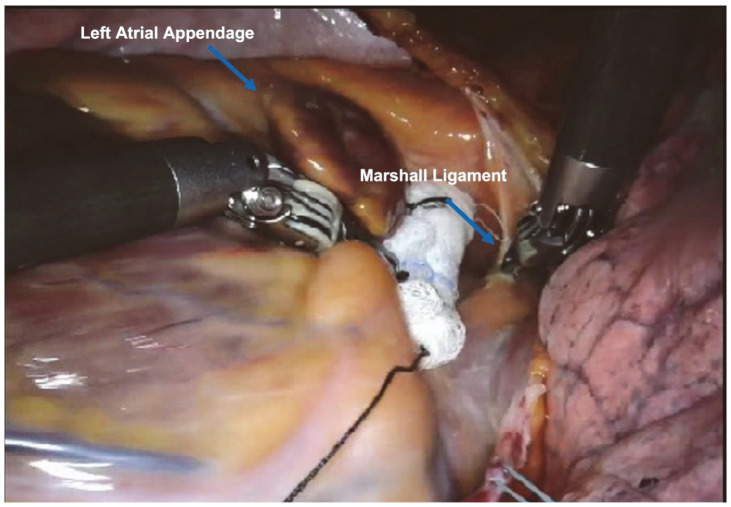
Marshall ligament dissection.

**Figure 3:**
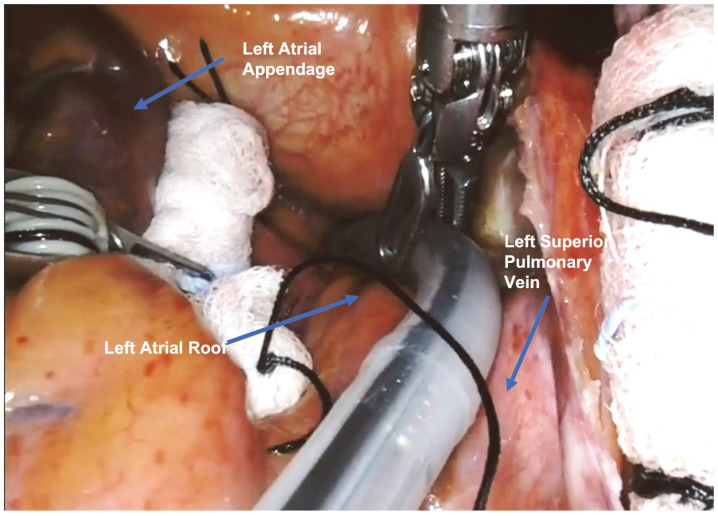
First ablation line on the left atrial roof, between the left and right superior pulmonary veins.

**Figure 4:**
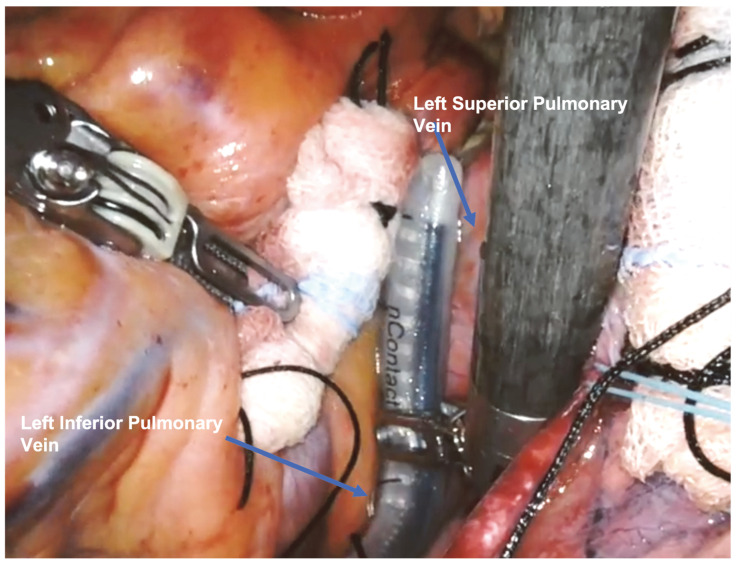
Second ablation line connecting the superior and inferior left pulmonary veins.

**Figure 5:**
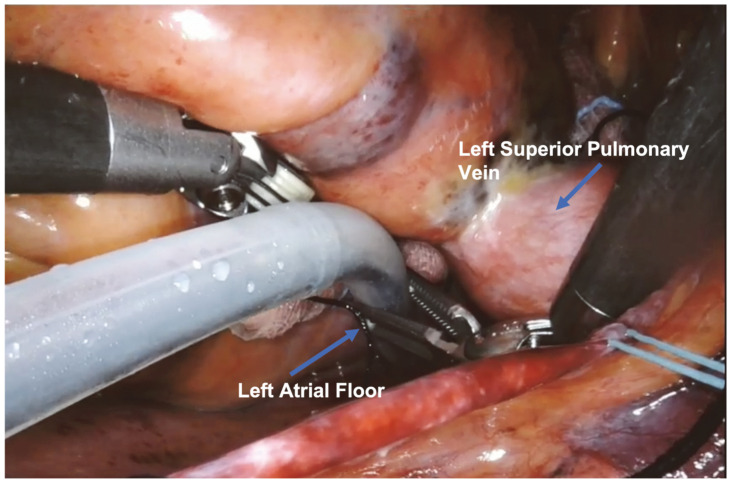
Third ablation line on the left atrial floor.

**Figure 6:**
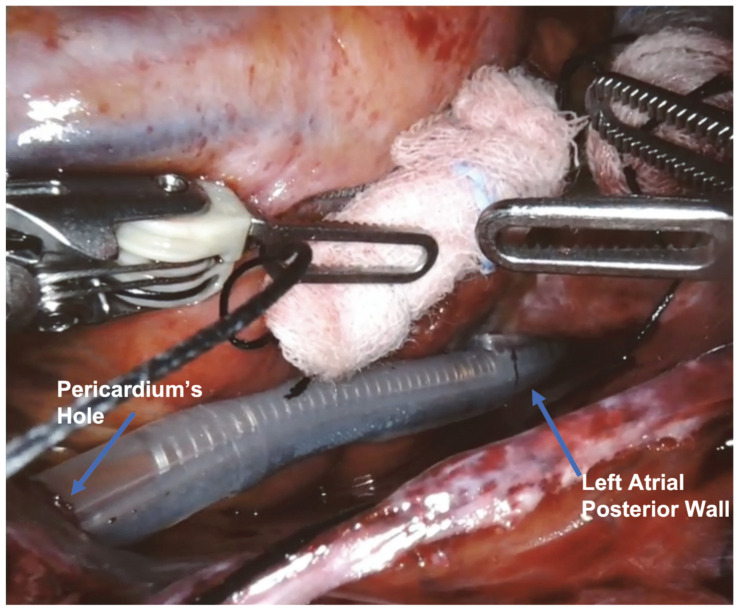
Ablation lines on the entire atrial posterior wall.

**Figure 7:**
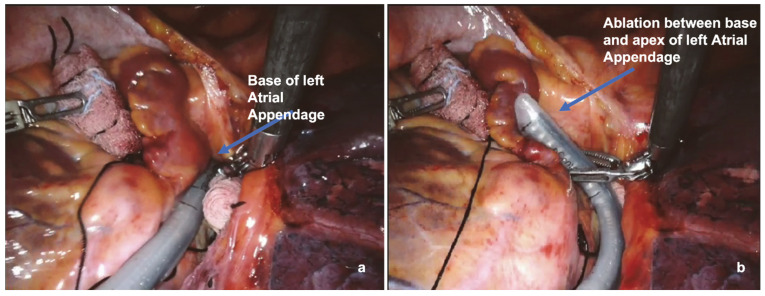
Ablation line at the base of the left atrial appendage (**a**) and between the base and apex of the LAA (**b**).

**Figure 8:**
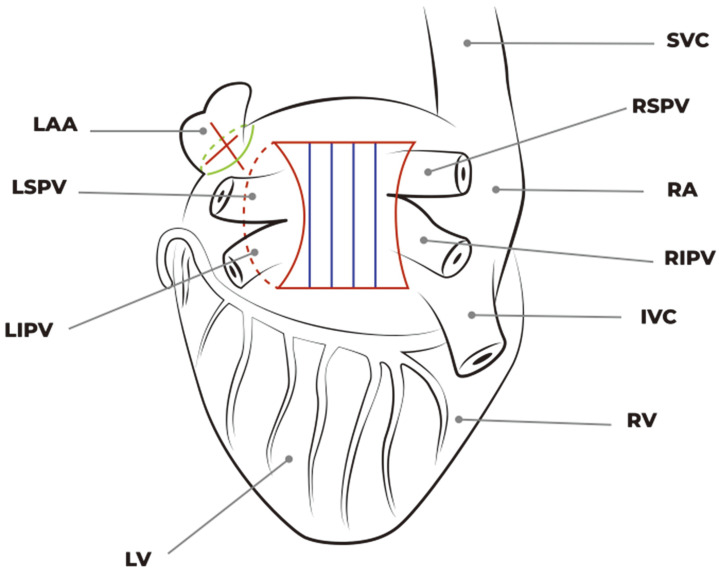
Complete set of ablation lines.

**Table 1:** Preoperative characteristic.

	Number of Patients = 18
Age (years)	64 ± 5
Female sex, *n* (%)	3 (17)
Family AF History, *n* (%)	4 (22)
Hypertension, *n* (%)	12 (67)
Diabetes, *n* (%)	4 (22)
Chronic obstructive pulmonary disease, *n* (%)	1 (6)
Dyslipidemia, *n* (%)	11 (61)
Coronary artery disease, *n* (%)	0 (0%)
Peripheral vascular disease, *n* (%)	0
Stroke, *n* (%)	0
Obesity, *n* (%)	9 (50)
CHA_2_DS_2_-VASc score	2 ± 1
Has-bled score	2 ± 1
Average AF duration (years)	4 ± 2
NYHA class I-II, *n* (%)	18 (100)
Previous CVE, *n* (%)	6 (33)
Previous PVI transcatheter ablation, *n* (%)	7 (39)
Previous transcatheter LA exclusion, *n* (%)	1(4)
Anatomical findings	
LAA morphology	
Cauliflower, *n* (%)	4 (22)
Chicken Wing, *n* (%)	6 (33)
Windsock, *n* (%)	3 (17)
Cactus, *n* (%)	5 (28)
MRI Left Atrial Volume (mL)	156 ± 37.7
Mean EF (%)	57 ± 6

**Table 2:** Intraoperative and postoperative characteristics.

	Number of Patients = 18
Concomitant AF ablation + LAA exclusion	100% (18)
Operative time (min) overall	142 ± 22
LAA clip size (mm)	
40	4
45	10
50	4
CPB required	0%
Convertion to full sternotomy or minithoracotomy	0%
Extubation in operative room	83% (15)
Mean mechanical ventilation time (Hours)	3 ± 1
Mean ICU time (hours)	16 ± 3
Postoperative stroke	0
Hospital mortality	0
Mean length of stay in hospital (days)	3 ± 1
Rhythm at discharge	
Sinusal rhythm	67% (12)
Atrial flutter	6% (1)
Atrial fibrillation	27% (5)

## Data Availability

Data are not published on publicly archive but are available after mail request to the corresponding author.
